# PIGK defects induce apoptosis in Purkinje cells and acceleration of neuroectodermal differentiation

**DOI:** 10.1038/s41419-024-07201-8

**Published:** 2024-11-09

**Authors:** Siyi Chen, Jiali You, Xiaowei Zhou, Yan Li, Fang Liu, Yanling Teng, Hua Teng, Yunlong Li, Desheng Liang, Zhuo Li, Lingqian Wu

**Affiliations:** 1 Center for Medical Genetics, Hunan Key Laboratory of Medical Genetics, MOE KeyLab of Rare Pediatric Diseases, School of Life Sciences, Central South University, Changsha, China; 2 Department of Medical Genetics, Hunan Jiahui Genetics Hospital, Changsha, China

**Keywords:** Genetics of the nervous system, Neurological disorders

## Abstract

Biallelic mutations in *PIGK* cause GPI biosynthesis defect 22 (GPIBD22), characterized with developmental delay, hypotonia, and cerebellar atrophy. The understanding of the underlying causes is limited due to the lack of suitable disease models. To address this gap, we generated a mouse model with PIGK deficits, specifically in Purkinje cells (Pcp2-cko) and an induced pluripotent stem cell (iPSC) model using the c.87dupT mutant (KI) found in GPIBD22 patients. Pcp2-cko mice demonstrated cerebellar atrophy, ataxia and progressive Purkinje cells loss which were accompanied by increased apoptosis and neuroinflammation. Similarly, KI iPSCs exhibited increased apoptosis and accelerated neural rosette formation, indicating that PIGK defects could impact early neural differentiation that confirmed by the RNA-Seq results of neural progenitor cells (NPCs). The increased apoptosis and accelerated NPC differentiation in KI iPSCs are associated with excessive unfolded protein response (UPR) pathway activation, and can be rescued by UPR pathway inhibitor. Our study reveals potential pathogenic mechanism of GPIBD22 and providing new insights into the therapeutic strategy for GPIBD.

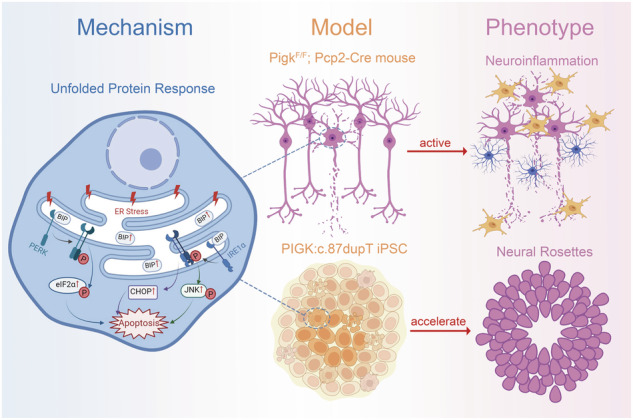

## Introduction

Glycosylphosphatidylinositol (GPI) modification is a highly conserved post-translational process in eukaryotic organisms that involves over 30 genes [1] participating in different steps in the GPI biosynthetic pathway [2, 3]. Defects in this pathway are associated with various pathologies known as GPI biosynthesis defects (GPIBDs) [4, 5]. Currently, OMIM documents 25 GPIBD subtypes associated with specific causative genes, rendering GPIBDs responsible for ~0.15% of all neurodevelopmental disorders [6]. Among these, biallelic mutations in *PIGK* can lead to GPIBD22, which is characterized by various neurological symptoms, particularly intellectual disability, hypotonia, epilepsy, and cerebellar atrophy [7, 8].

PIGK serves as the catalytic subunit of GPI-transamidase complex (GPI-T) facilitating the attachment of the GPI anchor to proteins [9, 10]. Its primary localization is in the endoplasmic reticulum (ER), whereas the other subunits are localized in both the ER and nuclear envelope (NE) [11]. In 2020, Nguyen et al. first reported that biallelic mutations in *PIGK* were associated with neurodevelopmental disorders in 12 patients from 9 families [7]. Patient-derived fibroblasts demonstrated reduced GPI-anchored proteins (GPI-APs). However, the precise pathogenic mechanism of PIGK in neurodevelopment in vivo remains unknown. To date, only two studies on GPIBD22 have been reported. Another one is about a Chinese GPIBD22 family which was reported by our group in 2021 [8]. Our patients manifested severe infantile encephalopathy with progressive global brain atrophy, which regressed before age four due to respiratory complications. They were noted to have the compound heterozygous variants c.87dupT (p.I30Yfs*10) and c.610 G > C (p.D204H) of *PIGK*. *Pigk* knock-in mice were generated to investigate the impact of these mutations on neurodevelopment. However, homozygosity for either mutation proved embryonically lethal. Owing to the lack of suitable disease models for GPIBD22, data on the pathophysiology of PIGK deficiency remain limited. Key questions regarding how *PIGK* mutations affect embryonic and neuronal development, as well as the specific pathological features and molecular mechanisms underlying GPIBD22, remain unanswered.

Considering the lethality of knock-in mice, we employed a conditional knockout strategy and reconstructed a *Pigk*^F/F^ mouse model to investigate the precise impact of PIGK on neurons, particularly those crucial for cerebellum function. Additionally, to further investigate the function of PIGK during early embryonic and neuronal development within the context of human genetics, we utilized the CRISPR-Cas9 system to introduce the previously documented patient-specific mutation *PIGK*: c.87dupT (p. I30Y fs*10) into iPSCs derived from a healthy individual. Next, we differentiated iPSCs into neural progenitor cell (NPC). Using iPSCs and mouse models, we investigated the specific pathogenic mechanisms underlying GPIBD22 induced by PIGK functional deficiency. This study provides novel insights into the prevention and treatment of such severe genetic diseases and will help to facilitate other types of GPIBD studies.

## Results

### cKO mice displayed delayed growth, ataxia, and progressive motor impairment

To examine the function of PIGK in cerebellar pathogenesis, we first investigated the expression of PIGK in the mouse brain and found that it was highly expressed in Purkinje cells of the cerebellum (Fig. S1A). Thus, we generated a *Pigk*^F/F^ mouse and crossed it with the Pcp2-cre transgenic line, widely utilized for Purkinje cell-specific gene knockout (Fig. S1B). PCR and Sanger sequencing validated the specificity of Cre expression and confirmed the presence of flanking exons 2–3 of *Pigk* (Fig. S1C, D). In subsequent statements, cKO referred to the conditional knockout mice (*Pigk*^F/F^; Pcp2 Cre^+^), while Wt denoted the control group (*Pigk*^F/F^; Pcp2 Cre^-^).

**Fig. 1:**
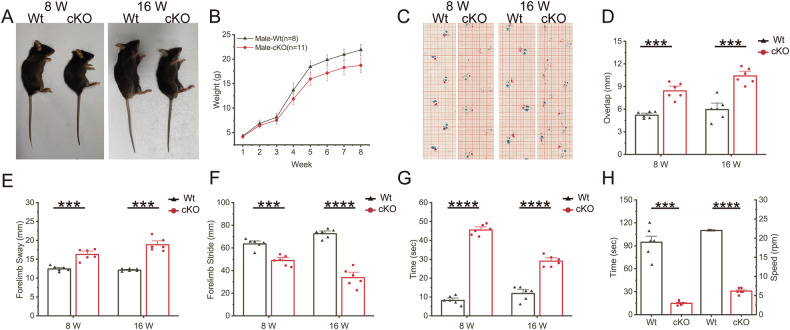
The Pigk^F/F^; Cre^+^ (cKO) mice exhibited progressive motor impairment. **A** Images depicting the bodies of Pigk^F/F^; Cre^+^ (Wt) and cKO mice at 8 and 16 weeks. **B** A comparison of weekly body weight from 1 to 8 weeks. Data are presented as mean values ± SD. **C** Representative traces of the footprint assay in Wt and cKO mice at 8 and 16 weeks. Quantification of the overlap length (**D**), the sway length (**E**), and the stride length (**F**) between the forelimbs of Wt and cKO mice at 8 and 16 weeks. **G** Quantification of latency to cross beam in Wt and cKO mice at 8 and 16 weeks. **H** The latency to fall and speed in the first fall of Wt and cKO mice at 8 weeks in rotarod testing. **D**–**H** N = 6 animals per genotype; data are presented as mean values + SEM; ***p < 0.001; ****p < 0.0001 (Welch’s *t*-test).

The cKO mice were viable and fertile, showing delayed growth and development after 2 weeks. Over time, the differences in body weight became more pronounced (Fig. 1A, B). By 8 weeks, we observed an unsteady gait in the cKO mice (Supplementary materials, Video 1). Footprint analysis, conducted to assess the walking patterns of the mice, involved calculating parameters such as overlap, sway, and stride (Fig. 1C and Fig. S2A). The cKO male mice showed increased overlap and sway along with reduced stride, indicative of ataxic gait (Fig. 1D–F). The differences were more pronounced in the 16-week-old mice. Similar results were observed in cKO female mice (Fig. S2B–D). To assess motor coordination, we conducted a balance beam experiment and a rotarod test. The cKO mice exhibited a significant increase in the time taken to traverse the beam (Fig. 1G and Fig. S2E–F). The results of the rotarod test at 8 weeks showed that the time and speed of the first fall in cKO mice were significantly lower than in Wt mice (Fig. 1H and Fig. S2G–H). At 16 weeks, the cKO mice could not complete the rotarod test (*Supplementary materials*, Video 2). These findings indicate that PIGK deficiency in Purkinje cells leads to delayed growth, ataxia, and progressive motor deficits.

### cKO mice exhibited cerebellar atrophy and progressive loss of Purkinje cells

**Fig. 2:**
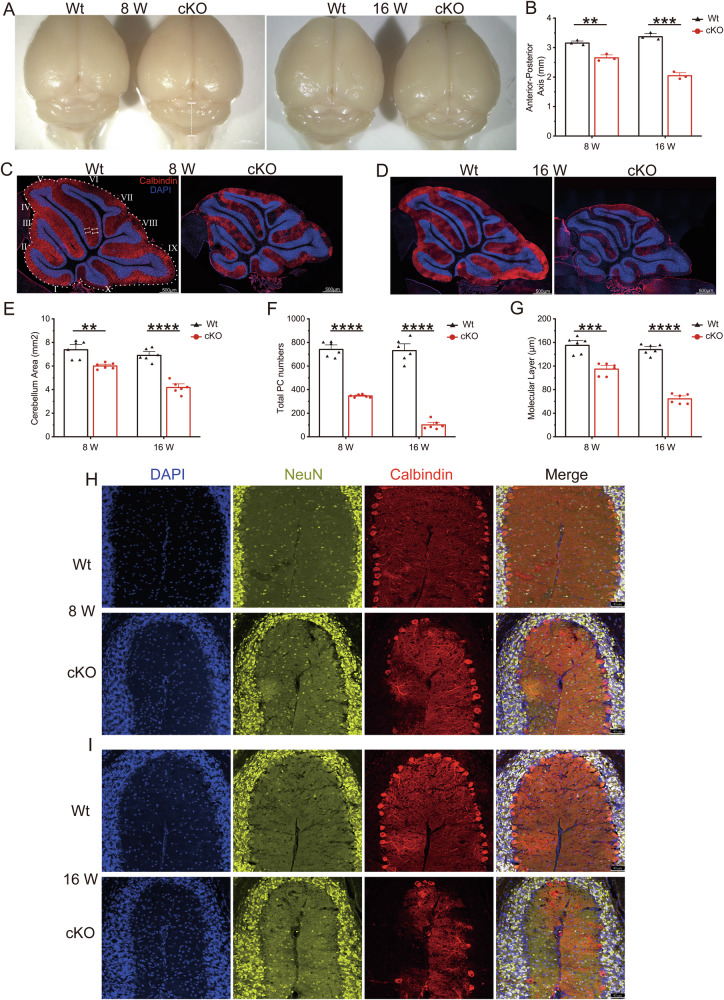
The cKO mice exhibited progressive loss of Purkinje cells. **A** Gross view of brains from Wt and cKO mice at 8 and 16 weeks, with white dotted lines representing the anterior–posterior axis. **B** Quantification of the anterior-posterior axis length, N = 3 animals per genotype; data are presented as mean values + SEM; **p < 0.01; ***p < 0.001 (Welch’s *t-*test). Immunostaining for calbindin on sagittal sections of Wt and cKO mice at 8 weeks (**C**) and 16 weeks (**D**). **E** Quantification of cerebellum area, measured by outlining the outer edge of midline sagittal sections of the cerebella. **F** Quantification of the total number of Purkinje cells in all cerebellar lobules. **G** Quantification of the molecular layer thickness in cerebellar lobulesV–VI. N = 3 animals per genotype, and two sections/animal; data are presented as mean values + SEM; **p < 0.01; ***p < 0.001; ****p < 0.0001 (Welch’s *t*-test). Coimmunostaining of NeuN and Calbindin with DAPI in cerebellar lobules II–III of Wt and cKO mice at 8 weeks (**H**) and 16 weeks (**I**).

Cerebellar samples were harvested at different developmental stages and stained along the sagittal plane for structural analysis to elucidate the pathogenic progression of the cerebellum in cKO mice. A panoramic view of the brain revealed progressive cerebellar atrophy in the cKO mice, with a significant reduction in the anterior–posterior axis. At 8 weeks, there was a 15% reduction, increasing to 40% by 16 weeks (Fig. 2A, B). Calbindin staining was employed to observe alterations in cerebellar lobules and Purkinje cells. In cKO mice, there was a notable reduction in the cerebellar sagittal plane area, with an approximately 11% reduction at 8 weeks that further diminished to approximately 40% by 16 weeks (Fig. 2C–E). Additionally, there was a significant decrease in Purkinje cell count, with 55% remaining at 8 weeks and 13.5% at 16 weeks (Fig. 2F). The molecular layer underwent substantial thinning, reducing to approximately 80% at 8 weeks and approximately 45% at 16 weeks (Fig. 2G). These findings indicate a progressive loss of Purkinje cells in cKO mice, consistent with the phenotypic progression of ataxia in mice.

To investigate the onset and the progress to later-stage of cerebellar atrophy and Purkinje cell loss, Calbindin staining was extended to include mice at the earlier (4 weeks) and later (16 months) stages. At 4 weeks, no significant differences were observed (Fig. S3A, C). However, at 16 months, pronounced cerebellar atrophy was observed, with an almost complete absence of residual Purkinje cells (Fig. S3B, D). These results suggest that the loss of Purkinje cells begins at least after 4 weeks, and this loss is the pathological basis for cerebellar atrophy and ataxia. Additionally, complete loss of Purkinje cells does not affect the lifespan of mice.

The other major neuron type in the cerebellum is NeuN-positive granule cells. Co-staining with NeuN and Calbindin revealed a reduced Purkinje cell count in cKO mice, accompanied by dendritic swelling and shortening (Fig. 2H–I). However, there were no significant changes in the size, quantity, or positioning of the granule cells (Fig. 2H–I), which suggests that the functional deficiency of PIGK in Purkinje cells leads to a progressive reduction in their count without affecting granule cells. Interestingly, an increasing abundance of DAPI signals was observed in the molecular layer of the cKO mice, intensifying over time (Fig. 2H–I and Fig. S3D). This phenomenon implies that the augmentation of these cells is associated with the progressive loss of Purkinje cells.

### cKO mice showed increased proliferation of glial cells and elevated apoptosis

**Fig. 3:**
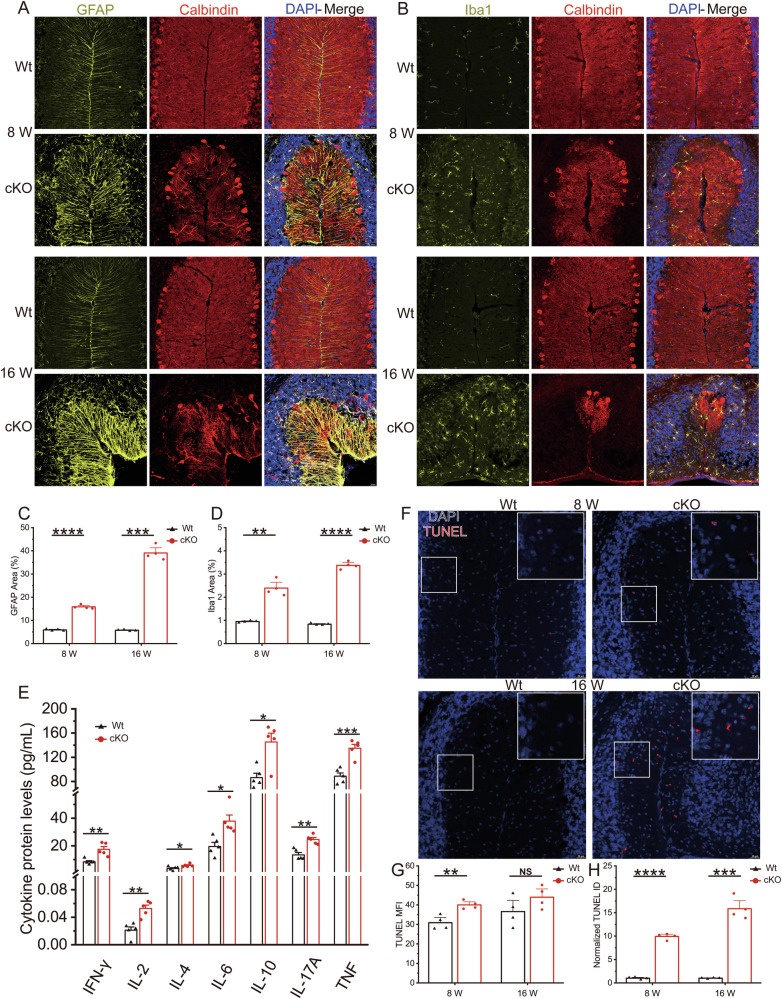
The cKO mice displayed increased apoptosis and microglial activation. **A** Coimmunostaining of GFAP and calbindin with DAPI in cerebellar lobules II-III of Wt and cKO mice at 8 weeks and 16 weeks. **B** Coimmunostaining of Iba1 and Calbindin with DAPI in cerebellar lobules II-III of Wt and cKO mice at 8 weeks and 16 weeks. **C** Quantification of GFAP fluorescence area fraction, representing the ratio of the fluorescent area region to the total area of the image. **D** Quantification of Iba1 fluorescence area fraction. **E** Quantification of cytokine protein levels. **F** Representative images of TUNEL staining in cerebellar lobules II-III of Wt and cKO mice at 8 and 16 weeks. The upper right corner of the image displays an enlarged view of the white box on the left side. Quantification of TUNEL mean fluorescence intensity (**G**) and total fluorescence intensity (**H**) normalized by Wt mice. N ≥ 4 animals per genotype; data are presented as mean values + SEM; NS indicates no significance, *p < 0.05; **p < 0.01; ***p < 0.001; ****p < 0.0001 (Welch’s *t*-test).

Considering the possible proliferation and activation of astrocytes and microglial cells to maintain the functionality of the remaining Purkinje cells, we hypothesized that the increased DAPI signals in cKO mice were proliferated and activated glial cells. GFAP staining revealed that the average GFAP area in 8-week-old cKO mice was approximately 15% and increased into 35% in 16 weeks compared with about 6% in Wt mice (Fig. 3A, C). IBA1 staining demonstrated the activation and proliferation of microglial cells in cKO mice, with approximately 2.3% activation at 8 weeks and increased into 3.4% at 16 weeks compared with approximately 1% in Wt mice (Fig. 3B, D). The activation and proliferation of glial cells can lead to the onset of neuroinflammation. Therefore, we assessed the expression levels of several cytokines. In cKO mice, the expression of IFN-γ, IL-2, IL-4, IL-6, IL-10, IL-17A, and TNF was all upregulated (Fig. 3E). These alterations in inflammation-related cytokines collectively indicate that the cerebellum of cKO mice undergoes significant neuroinflammation, with a widespread activation of the immune response.

Drawing from observations in our previous zebrafish model [8], where widespread neuronal apoptosis was observed, and aiming to explore the reasons for Purkinje cell loss further, we conducted TUNEL staining on frozen cerebellar sections. The results revealed a 10-fold increase in TUNEL signals in 8-week-old cKO mice and an approximately 15-fold increase in 16-week-old cKO mice (Fig. 3F–H). These findings indicate that Purkinje cells undergo apoptosis in cKO mice over time while astrocytes and microglial cells are activated to eliminate apoptotic Purkinje cells and protect the remaining Purkinje cells.

### KI iPSCs manifested defects in GPI-AP biosynthesis and increased apoptosis

Our previous knock-in mice experienced early embryonic death, indicating the crucial role of Pigk in embryonic development. However, our cKO mice start expressing Cre after birth, and considering the genetic background differences between humans and mice, we used iPSCs to investigate the effect of PIGK on the early development of human embryos. We utilized the CRISPR-Cas9 system to introduce the *PIGK*: c.87dupT (p.I30Yfs*10) mutation into iPSCs derived from healthy individuals. Sanger sequencing was employed to confirm the establishment of an iPSC model harboring the homozygous *PIGK*: c.87dupT mutation (KI) (Fig. S4A). This mutation did not affect mRNA expression but caused reduced protein expression (Fig. S4B, C), consistent with previous findings in lymphocyte cell lines derived from patients. Off-target verification of the three selected sites revealed no off-target events (Fig. S4D). The KI iPSCs exhibited normal karyotype and clone morphology (Fig. S4E, F).

**Fig. 4:**
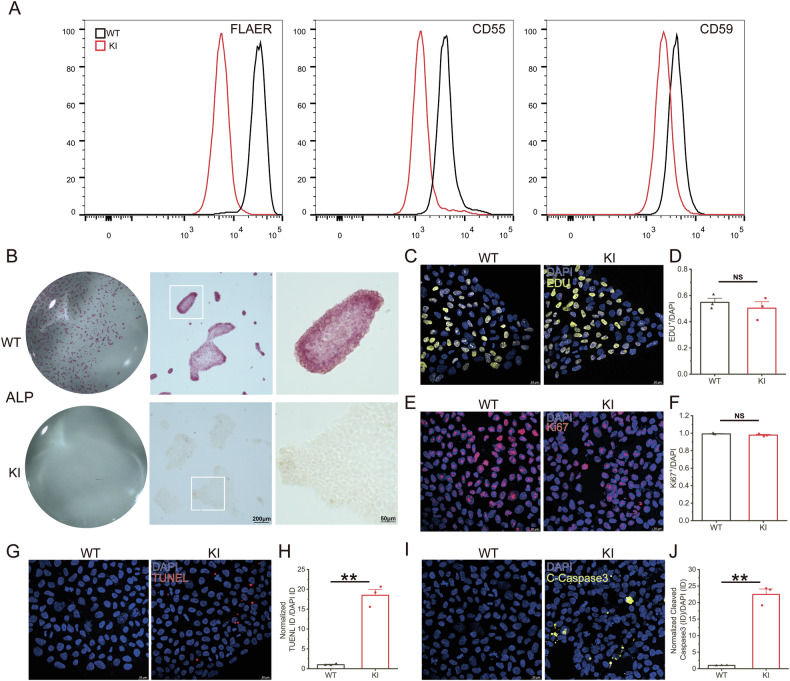
The PIGK: c.87dupT (KI) iPSCs manifested defects in GPI-AP biosynthesis and increased apoptosis. **A** Representative flow cytometry analysis of WT and KI iPSCs demonstrating the relative expression of GPI-APs stained with FLAER, CD55, and CD59, respectively. **B** Alkaline phosphatase (ALP) staining. ALP positivity is pink-purple, and ALP negativity is light yellow. The right images show magnified views of the white box area. Representative images of Ki67 staining (**C**) and quantification of the ratio of Ki67-positive cell numbers to total DAPI^+^ cells (**D**) in WT and KI iPSCs. Representative images of EDU staining (**E**) and quantification of the ratio of EDU-positive cell numbers to total DAPI^+^ cells (**F**) in WT and KI iPSCs. Representative images of TUNEL staining (**G**) and quantification of the ratio of TUNEL total fluorescence intensity (ID) to total DAPI ID and normalized by WT iPSCs (**H**). Representative images of cleaved caspase3 immunostaining (**I**) and quantification of the ratio of cleaved caspase3 ID to total DAPI ID, normalized by WT iPSCs (**J**). N = 3 independent experiments; data are presented as mean values + SEM; NS indicates no significance; **p < 0.01 (Welch’s t-test).

PIGK deficiency hinders GPI transamidase function, thereby affecting GPI-AP cell membrane localization. Flow cytometry revealed decreased FLAER signals in KI iPSCs, indicating an overall reduction in GPI-AP expression (Fig. 4A). Both CD55 and CD59 showed reduced expression, although the decrease in CD59 expression was not statistically significant (Fig. 4A). Alkaline phosphatase (ALP), a GPI-AP highly expressed in stem cells and as a Pluripotency marker, was absent in the KI iPSCs (Fig. 4B). However, immunofluorescence staining for NANOG and SOX2, which are commonly used to determine iPSC pluripotency, showed no significant differences between the KI and WT iPSCs (Fig. S4G). Next, we investigated the effects of PIGK deficiency on iPSC proliferation and apoptosis. EDU and Ki67 staining demonstrated no difference in proliferation between KI and WT iPSCs (Fig. 4C–F). We employed TUNEL and Cleaved-Caspase3 staining to explore changes in apoptosis in KI iPSCs and revealed multiple strong signals in KI iPSCs, with a normalized increase of approximately 19-fold (Fig. 4G–H). Cleaved-Caspase3, representing the activated form of Caspase3 detectable only in apoptotic cells, showed an approximately 23-fold increase in fluorescence intensity in KI iPSCs (Fig. 4I–J). These findings demonstrate that PIGK functional deficiency affects GPI-AP biosynthesis and may influence the partial pluripotency of iPSCs but does not affect their self-renewal capacity. Moreover, PIGK defects may increase cell apoptosis, consistent with observations in cKO mouse’s Purkinje cells.

### The UPR-mediated apoptosis pathway was activated in cKO mice and KI iPSCs

**Fig. 5:**
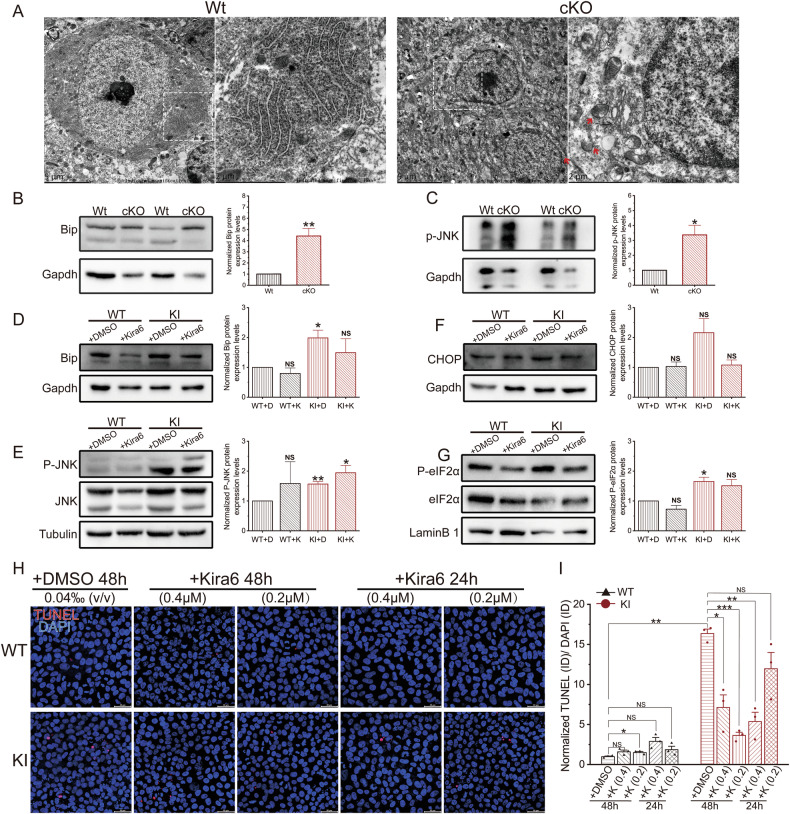
Activation of ER stress-related UPR pathways in cKO mice and KI iPSCs. **A** Representative electron microscopy image of Purkinje cells in Wt and cKO mice at 8 weeks, with a magnified detail (white dashed box) on the right. The red arrows indicate swollen endoplasmic reticulum in cKO mice. Western blot and quantification of BIP levels (**B**) and p-JNK levels (**C**) in Wt and cKO mice. N = 4 animals per genotype; data are presented as mean values + SEM; *p < 0.05; **p < 0.01 (Welch’s t-test). Western blot and quantification of BIP levels (**D**), P-JNK levels (**E**), CHOP levels (**F**), and P-EIF2α levels (**G**) in WT and KI iPSCs. The WT + D or KI + D group represents treatment with 0.05% DMSO for 12 h, while the WT + K or KI + K group represents treatment with 0.5 μM Kira6 for 12 h. N = 5 independent experiments; data are presented as mean values + SEM; NS indicates no significance; *p < 0.05; **p < 0.01 (Welch’s *t*-test). Representative images of TUNEL staining (**H**) and quantification of the ratio of TUNEL ID to DAPI ID (**I**). N = 3 independent experiments; data are presented as mean values + SEM; NS indicates no significance; *p < 0.05; **p < 0.01; ***p < 0.01 (Welch’s *t*-test).

To investigate the underlying causes of apoptosis, considering that GPI synthesis occurs in the ER, we hypothesized that PIGK deficiency disrupts ER homeostasis and leads to the activation of the unfolded protein response (UPR)–apoptosis pathway. During ER stress, Bip is recruited to activate three UPR branches: IRE1α, PERK, and ATF6. The classic IRE1α or PERK-eIF2α UPR branch ultimately induces apoptosis by activating the CHOP or JNK signaling pathway [12, 13]. Electron microscopy, employed to observe ER morphology in Purkinje cells, revealed ER swelling and ribosomal detachment within these cells in cKO mice, indicative of ER damage. Additionally, deep nuclear staining and indistinct cell membranes suggested that the Purkinje cells were in the late degeneration stage (Fig. 5A). Western blot analysis revealed significantly increased Bip expression (Fig. 5B) and markedly elevated levels of phosphorylated JNK (P-JNK) (Fig. 5C) in the cKO mice, indicating activation of the IRE1α-mediated apoptotic pathway. Bip and P-JNK levels were also elevated in KI iPSCs (Fig. 5D–E).

To determine whether IRE1α inhibitors can relieve ER stress, we used Kira6 in KI and WT iPSCs. Kira6 is a small-molecule inhibitor that targets IRE1α, and can help mitigate cellular stress and apoptosis by reducing UPR hyperactivation [14–16]. Upon treatment, Bip expression doubled in the KI + DMSO group compared with that in the WT + DMSO group but decreased in the KI + Kira6 treatment group (Fig. 5D). However, Kira6 treatment increased the P-JNK expression levels in both KI and WT iPSCs (Fig. 5E). Moreover, another pro-apoptotic protein, CHOP, increased in the KI + DMSO group, and its normal level was restored in the KI + Kira6 group (Fig. 5F). These results indicate that the IRE1α UPR pathway was activated and that Kira6 could partially alleviate ER stress. The activation status of the PERK branch was explored by assessing the levels of phosphorylated eIF2α (P-eIF2α). The expression level increased in the KI + DMSO group, but the Kira6 treatment did not alleviate this increase (Fig. 5G), suggesting that PERK was also activated in KI cells.

We subsequently investigated whether Kira6 treatment could alleviate apoptosis in KI iPSCs. TUNEL assay results indicated that treatment with 0.4 μM Kira6 for both 24 hours and 48 hours significantly reduced apoptosis in KI cells. At a concentration of 0.2 μM, treatment for 48 hours effectively reduced apoptosis while 24 h treated was not statistically significant (Fig. 5H–I). Cleaved caspase-3 staining showed similar results (Fig. S5A-B). Kira6 reduces IRE1α activity in a dose-dependent manner, but it also exhibits significant cytotoxicity at low concentrations in Neuro2a cells [17]. In WT iPSCs, we also observed an increase in apoptosis following prolonged or high-concentration Kira6 treatment. These findings collectively indicate that the functional deficiency of PIGK promotes cellular apoptosis through UPR-related pathways and could partially rescued by Kira6 treatment.

### KI iPSCs displayed acceleration of neural rosette formation

**Fig. 6:**
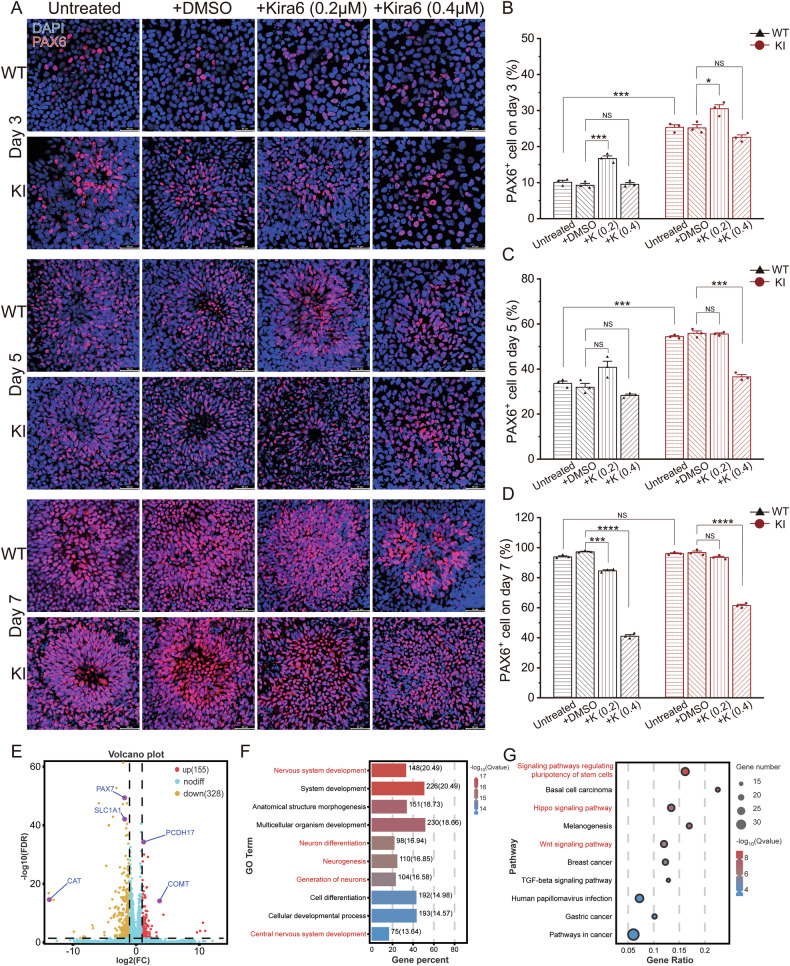
The KI iPSCs underwent premature differentiation into neural progenitor cells (NPCs). **A** Representative images of immunofluorescence staining for PAX6 on day3, 5 and 7. Quantification of the ratio of PAX6-positive cell numbers to total DAPI^+^ cells in WT and KI cells on day3 (**B**), 5 (**C**) and 7 (**D**). N = 3 independent experiments; data are presented as mean values + SEM; NS indicates no significance; ***p < 0.001; ****p < 0.0001 (Welch’s *t*-test). **E** Volcano plot of differentially expressed genes from RNA sequencing of WT and KI NPC on day7. The criteria for selecting differentially expressed genes are |Log_2_Fold Change | >1 and P_adjusted_ (FDR) < 0.05. **F** The top 10 significant GO terms of all differentially expressed genes. Terms related to neuron development are presented in red front. **G** The top 10 significant Kegg Pathway of all differentially expressed genes. Terms related to neuron development are presented in red front.

The dynamic regulation of the UPR pathway is closely associated with neurogenesis [18]. Therefore, we differentiated iPSCs into NPCs to investigate the impact of UPR activation on early neurogenesis. During the differentiation, we observed that KI cells exhibited columnar cell morphology and rosette-like aggregation as early as day3 (Fig. S6A). Upon passaging on day 7, we noticed fewer KI cells adhering compared to WT cells on day 8 (Fig. S6A), indicating potential impairment in adhesion after KI cell differentiation into NPCs. To validate whether KI cells undergo premature differentiation, we performed PAX6 (early NPC marker) and SOX1 (NPC marker) staining on day 3, 5, and 7. On days 3, 5, and 7, the percentage of SOX1-positive cells in KI was approximately 22%, 35%, and 83%, respectively, all of which were significantly higher than in WT (Fig. S6B-C). As for the percentage of PAX6-positive cells, KI cells exhibited significantly higher levels than WT on days 3 and 5 (Fig. 6A–C). However, by day 7, both untreated groups reached over 90%, with no significant difference (Fig. 6D). These results suggest that the KI iPSCs showed acceleration of neural rosette formation.

To investigate whether the accelerated NPC differentiation observed in KI is due to excessive UPR activation, we added 0.2 μM and 0.4 μM Kira6 to the differentiation medium. Unexpectedly, under 0.2 μM Kira6 treatment, the percentage of PAX6-positive cells increased in both KI and WT cells on day 3 and there were no changes in KI cells on days 5 and 7 compared to DMSO treatment (Fig. 6B–D). Surprisingly, at a concentration of 0.4 μM, the percentage of PAX6-positive cells in KI cells showed a decreasing trend on day 3, and by day 5, it had declined to levels comparable to WT cells. By day 7, the difference became more pronounced (Fig. 6B–D). These results suggest that the accelerated differentiation of KI iPSCs into NPCs is driven by UPR pathway activation and could be reversed by Kira6.

To obtain a broader and unbiased understanding of the differences between the WT and KI NPCs that may impact subsequent neurogenesis, we performed RNA-Seq on NPCs at day 7 when the percentage of PAX6-positive cells was more than 90%. Gene differential analysis (|Log_2_Fold Change | >1, P_adjusted_ < 0.05) revealed 328 downregulated genes and 155 upregulated genes (Fig. 6E). Among them, *PAX7*, *SLC1A1* and *CAT* were significantly downregulated genes, while *PCDH17* and *COMT* were upregulated (Fig. 6E). These genes are crucial for maintaining the functionality of the nervous system. Ontology (GO) analysis identified an enrichment for biological process including nervous system development, neuron differentiation and neurogenesis (Fig. 6F). The KEGG pathway analysis enriched in signaling pathways regulating pluripotency of stem cells, Hippo signaling pathway and Wnt signaling pathway (Fig. 6G). These signaling pathways play important roles in nervous system development. The RNA-Seq provided a transcriptional-level validation that KI iPSCs exhibited abnormal neurodevelopmental programs, potentially affecting the subsequent differentiation and function of neurons.

## Discussion

PIGK is essential for neuronal function; however, suitable models to study its role in vivo remains lacking. In this study, we constructed a Pigk–Purkinje cell-specific knockout mouse model to investigate pathological alterations in the cerebellum and the underlying molecular mechanisms of GPIBD22. The cKO mice exhibited delayed growth and development, progressive cerebellar atrophy, motor impairment, and ataxia, all of which were consistent with the main phenotypes observed in GPIBD22 patients. Our cKO mice, with prolonged survival and significant phenotypes, such as cerebellar atrophy, are valuable mammalian models for investigating GPIBD22. We observed no obvious differences in cKO mice Purkinje cells at 4 weeks; however, by 8 weeks, Purkinje cell loss became evident, progressing over time. Almost no surviving Purkinje cells were observed at 16 months.

This progressive loss correlates with worsening phenotypes, such as motor dysfunction and ataxia. Apoptotic signals gradually increased in cKO mice, synchronizing with Purkinje cell loss. Currently, few reports exist on viable GPIBDs mouse models involving only *Piga*, *Pigv*, *Pigo*, *Pign*, *Pgap1* and *Pgap2* [19–23]. *Piga*^flox/WT^; Nestin-Cre mosaic cKO female mice exhibited cerebellar atrophy, motor disturbances, and dendritic branching abnormalities in Purkinje cells [19]. Severe motor dysfunction phenotypes were observed in *Pigv*^A341E^ and *Pigo*^T130N^ mice; however, the cerebellum exhibited no abnormalities [20, 21]. These results suggest that compared with other genes involved in GPI-AP synthesis, *Pigk* and *Piga* are more crucial for Purkinje cells. Our mouse model provides initial evidence for the importance of PIGK in Purkinje cell development and proposes Purkinje cell apoptosis as the main pathological mechanism underlying GPIBD22 cerebellar atrophy. These findings offer insights for selecting intervention time points and improving indicators for future therapeutic strategies.

During Purkinje cell loss, GFAP and IBA1 staining in cKO mice of different ages increased, indicating astrocyte and microglia proliferation, a sign of neuroinflammation which was confirmed by changes in inflammation-related cytokines. At 8 weeks, the areas of GFAP and IBA1 staining increased and became more pronounced by 16 weeks. This phenomenon suggests that the inflammatory response in cKO mice intensified with Purkinje cell loss. Currently, studies on brain pathology staining in patients with GPIBD are limited; therefore, whether these patients develop neuroinflammation remains unclear. However, considering the prevalence of neuroinflammation in many neurodegenerative and glycosylation disorders [24–26], the neuroinflammation observed in cKO mice may also occur in patients with GPIBDs. Interestingly, magnetic resonance imaging (MRI) of one GPIBD21 patient’s brain revealed a small focal periventricular gliosis, while the contribution of gliosis to the phenotype remains unknown [27]. Neuroinflammation both benefits and adversely affects the nervous system. Targeted anti-inflammatory and neuroprotective therapies, which have shown promise for neurodegenerative diseases [28, 29], are potential treatment strategies for improving GPIBD symptoms. However, an in-depth understanding of the immune processes involved in the progression of these diseases remains necessary.

Apoptosis was significantly increased in KI iPSCs, whereas proliferation showed no significant changes, indicating that pathological alterations in apoptosis due to PIGK deficiency are commonly observed across various organisms. Besides, the apoptosis also occurs in neural crest cells and cranial neuroepithelium of *Pgap2* mutant mouse embryos [23]. Elucidating the mechanisms underlying apoptosis will provide valuable insights into the molecular changes associated with all GPIBDs. Our experiments indicated that in both cKO mice and KI iPSCs, ER stress, and the UPR pathway were activated, which could be partially alleviated by Kira6 treatment. Additionally, Kira6 treatment partially alleviated apoptosis in KI iPSCs. Currently, there is no research characterizing the relationship between the UPR pathway and GPIBD22. The UPR pathway participates in various physiological processes, including brain development, neuronal physiology, behavior, and pathological changes observed in neurodegenerative diseases [30, 31]. Our study suggests that PIGK deficiency triggers the UPR pathway and subsequent apoptosis, serving as the molecular mechanism underlying GPIBD22. Targeted alterations in specific UPR components, utilizing small molecules and gene therapies, may provide new avenues for impeding or preventing disease progression [32, 33].

When we differentiated iPSCs into NPCs, the KI iPSCs could successfully differentiate to NPCs, but accelerated neural rosette formation manifested with the appearance of columnar PAX6+ cells and clusters at Day3. The acceleration would impact the time-course and order of neural differentiation and lead to an imbalance in the differentiation of neural cell types, resulting in developmental disorders. In Down syndrome, the acceleration also occurred in the differentiation process of patient’s iPCSs towards NPCs [34]. Our study revealed that this accelerated differentiation is related to excessive UPR activation, and treatment with 0.4 μM Kira6 effectively slowed this process. In studies on Zika virus-induced microcephaly, UPR activation was also found to promote direct neurogenesis, and Kira6 treatment could effectively reverse the increase of Nestin and PAX6 positive NPCs [35, 36]. These findings suggest that excessive activation of the UPR pathway may lead to the acceleration of early neurogenesis in many neurodevelopmental disorders. Thus, maintaining UPR pathway activity at normal levels could serve as a potential therapeutic target.

Our RNA-Seq analysis of NPCs at Day7 revealed that differentially expressed genes are primarily concentrated in neural system development. Among the downregulated genes, *PAX7* encoding a transcription factor is required for neural crest formation and biallelic variants in which are a genetic cause of myopathy characterized by hypotonia [37, 38]. *CAT*, encoding catalase, downregulation of which may lead to increased oxidative stress, affecting apoptosis and NPC migration [39]. *PCDH17* and *COMT* are the significantly upregulated genes. PCDH17, belonging to the protocadherin family, are implicated in synaptic function in the central nervous system. *PCDH17* overexpression promotes the mobility of synaptic vesicle clusters along axons [40]. COMT, one of the major mammalian enzymes involved in catecholamine metabolism and degradation, with increased expression and enzymatic activity observed in Down syndrome children and model mice [41, 42]. The RNA-Seq results indicate that these differentially expressed genes are closely associated with diseases such as myopathy, epilepsy, and Down syndrome, suggesting a potential correlation with the phenotype of GPIBD22. This also suggests that in the subsequent differentiation of NPC into specific neurons, attention should be paid to excitatory neurons as well as aspects such as neuronal migration and synaptic transmission.

Our cKO mice and KI iPSCs are suitable disease models for studying GPIBD22. Our cKO mice effectively mimicked the severe phenotypes observed in patients with GPIBD22, such as cerebellar atrophy and ataxia. Additionally, Purkinje cell loss and neuroinflammation onset are pathological features of cerebellar dysfunction. These new pathological findings provide clear therapeutic indicators for GPIBD22. Our in vivo and in vitro experiments collectively suggest that increased apoptosis due to UPR pathway activation is a potential molecular mechanism underlying GPIBD22. Our study elucidates the pathological progression and mechanisms of GPIBD22, offering new insights for treating GPIBD.

## Materials and methods

### Animals

*Pigk*^Flox/-^ mice were generated by GemPharmatech Co., Ltd. (Nanjing, China). using CRISPR/Cas9, while *Pcp2*-Cre mice were purchased from Cyagen Biosciences, Inc. (Guangzhou, China). *Pigk*^F/F^ mice were crossed with the *Pcp2*-Cre mice to generate a Purkinje cell–specific Pigk knockout mouse model named cKO, with *Pigk*^F/F^ mice serving as the control (Wt). All mice were maintained on a C57BL/6 background and housed in a barrier facility. Genotypes were identified through PCR amplification using specific primers: *Pigk* Flox site with Pigk-5’F/5’R and Cre recombinase with Pcp2-Cre F/R (*Supplementary materials*, Table S1). General PCR amplification was conducted to verify *Pigk* gene knockout efficiency using Pigk-5’F/3’R primers (*Supplementary materials*, Table S1). Blinding and randomization were not performed in this study.

### Generation of *PIGK*:c.87dupT iPSCs

The *PIGK*:c.87dupT iPSC model was created through gene editing of control human iPSCs which were derived from the urine cells of a healthy male donor. Plasmids carrying sgRNAs and ss ODN were introduced into iPSCs using the Nucleofector II set (Lonza, Basel, Switzerland) in the B016 program. After electroporation, single-cell seeding was performed at a low density. DNA was extracted from iPSC clones and subjected to Sanger sequencing (Sangon Biotech, Shanghia, China). Clones with homozygous mutations in *PIGK* were identified as positive clones (KI), while clones with wild-type *PIGK* were designated as control ancestors (WT). Subsequently, the obtained cells were subjected to another round of single-cell selection. The iPSCs were cultured in mTeSR Plus medium (STEMCELL Technologies Inc., Vancouver, Canada) on a Matrigel-coated plate, maintained at 37 °C in a humidified incubator with 5% CO2, and the medium was replaced daily. The primer sequences used for editing are listed in (Supplementary materials, Table S1). The cell lines used in this study were not authenticated and mycoplasma contamination was tested monthly using PCR.

### Statistical analysis

ImageJ was used to analyze the parameters such as area, quantity, fluorescence intensity of immunofluorescence images, and grayscale values of WB bands. Data analysis was performed using Origin2022. Statistical analyses comparing two groups were conducted using the Welch t-test. Data are presented as mean values + SEM; NS indicants no significance; *p < 0.05; **p < 0.01; ***p < 0.001; ****p < 0.0001.

## Supplementary information

Supplementary-movie-1

Supplementary-movie-2

Supplementary information

## Data Availability

The raw sequence data of RNA-seq on NPCs during the current study are available in the in the NCBI Sequence Read Archive (SRA) database. BioProject: PRJNA1108057.
