# Supplementary material for: PIGK defects induce apoptosis in Purkinje cells and acceleration of neuroectodermal differentiation
Source: Cell Death Dis. 2024 Nov 9;15(11):808. doi: 10.1038/s41419-024-07201-8 (PMC11550446; doi:10.1038/s41419-024-07201-8)
Supplement: Supplementary file 3 — Supplementary information [file 41419_2024_7201_MOESM3_ESM.pdf]

Supplemetary information

PIGK defects induce apoptosis in Purkinje cells and acceleration of neuroectodermal differentiation.

This file includes:

Supplementay materials and methods

Supplementary Figuer1-6

Supplementary movie1-2

Supplementary Table 1-2

Full and uncropped Western blots

Supplementary materials and methods

Behavioral testing

Behavioral experiments on mice were conducted between 12:00 and 18:00 at 8 and 16 weeks of age. Each genotype–sex combination comprised six mice.

Footprint analysis

The hindpaws and forepaws of the mice were painted with nontoxic blue and red watercolors, respectively. The mice then walked through a tunnel (75 cm long, 5 cm wide) lined with white paper on the floor. Pawprint patterns were analyzed for stride length, sway, and the distance between the front and hind pawprints on each side. Clear and continuous 25-cm prints were measured.

Balance beam test

Mice were placed on a balance beam (12 mm wide) elevated 50 cm above the ground, with one end positioned over a dark box and a hole located 100 cm away from the starting point. The first 2 days were the training period, while the third day was the testing period. The time taken by mice to reach the dark box was recorded.

Rotarod test

Mice were placed on a rotarod apparatus, which accelerated uniformly from 0 to 22 revolutions per minute (rpm) within 1 min, followed by continuous rotation at 22 rpm for 1 min. The first 3 days were the training period, with each mouse undergoing 6 min of training. On day 4, the mice were tested and the time and rotational speed at which they fell off the rotarod for the first time were recorded.

Immunofluorescence of the cerebellum

Mice were anesthetized with an intraperitoneal injection of 1.25% tribromoethanol (0.2 ml/10 g) based on body weight and sacrificed with transcardial perfusion with phosphate-buffered saline (PBS), followed by 4% paraformaldehyde (PFA) in PBS. The cerebellum was removed and fixed in 4% PFA in PBS for 24 h and subsequently dehydrated in 20% or 30% sucrose solution for 24 h each at 4 °C. Cryosections (20 µm thick; model CM 1860, Leica) of embedded O.C.T. (Sakura TissueTek, CA, USA) were generated. For each genotype, at least two histological sections at the middle sagittal

level from four different animals were analyzed for immunostaining, and confocal optical sections were obtained. Frozen sagittal sections were cut and incubated in a blocking solution for 1 h at 25°C. After rinsing with PBS, the sections were first incubated with primary antibodies overnight at 4°C and then with secondary antibodies for 1 h at 25°C. All antibody dilutions utilized for immunohistochemistry are detailed in (*Supplementary materials*, Table S2) and were diluted in PBS containing 0.2% Triton X-100 and 5% BSA.

Flow cytometry

The iPSCs were harvested at 80–90% confluency and stained with Alexa 488-conjugated aerolysin (FLAER; CedarLane Laboratories, Hornby, Ont., Canada), fluorescein isothiocyanate (FITC)-conjugated mouse antihuman CD55, and CD59 (BD PharMingen, San Diego, CA, USA) monoclonal antibodies for 0.5 h on ice and analyzed using the BD FACSCanto II system (BD Biosciences, USA) and FlowJo software.

EDU labeling

Following the guidelines provided by the BeyoClick™ EdU Cell Proliferation Kit with Alexa Fluor 488 (Beyotime, Shanghai, China), cells were seeded onto coverslips and cultured in a medium containing a final concentration of 10 μM EDU for 30 min. Subsequently, the cells were fixed and washed, followed by the sequential preparation of the reaction solution, which was incubated at 25°C in the dark for 30 min. After removing the reaction solution, the cells were washed three times with PBS and labeled with DAPI for nuclear staining.

TUNEL staining

Based on the instructions of the One-Step TUNEL Apoptosis Assay Kit (Beyotime, Shanghai, China), the cells seeded on coverslips or frozen tissue sections were fixed with 4% PFA for 30 min. After two washes with PBS, the cells were permeabilized with 0.4% Triton X-100 for 5 min. TUNEL detection solution was prepared and applied to the samples, which were incubated at 25°C in the dark for 1 h. Subsequently, the samples were washed three times with PBS, followed by nuclear staining with DAPI.

Western Blot

For mouse cerebellum analysis, the tissue was harvested, minced, and homogenized using a tissue homogenizer in an SDS lysis buffer (Beyotime, Shanghai, China). The buffer contained 1 mM PMSF (Beyotime, Shanghai, China), 1 × protease inhibitor cocktail (Sigma-Aldrich), and 1 × phosphatase inhibitor (Solarbio, Beijing, China). The iPSCs were harvested and lysed using RIPA lysis buffer (Beyotime, shanghai, China) supplemented with 1 mM PMSF, 1 × protease inhibitor cocktail, and 1 × phosphatase inhibitor. Protein concentrations were determined using a BCA protein assay kit (Thermo Fisher Scientific, Rockford, USA) following the manufacturer's instructions. Equal amounts of protein samples were loaded onto 10% sodium dodecyl sulfate–polyacrylamide gels and electrophoresed. Proteins were transferred from the gel to a polyvinylidene fluoride (PVDF) membrane (Merck Millipore, Burlington, MA, USA) using a wet transfer apparatus according to standard protocols. The PVDF membrane was blocked with 5% (w/v) skim milk powder in PBS containing 0.1% Tween-20 (PBST) for 1 h at 25°C with gentle shaking. After incubation with primary and secondary antibodies, the proteins were detected using the SuperSignal West Pico PLUS Chemiluminescent Substrate (Thermo Fisher Scientific, Rockford, USA). The antibodies used for western blotting are listed in (*Supplementary materials*, Table S2).

Transmission electron microscopy (TEM)

The cerebellum was sliced into 1 × 1 × 3 mm^3 sections and double-fixed in a 2.5% glutaraldehyde solution with Miller's phosphate buffer (pH = 7.3). The samples were then washed three times at 10-min intervals with Millonig's phosphate buffer and incubated for 1 h in 1% osmium tetroxide. After another three washes at 10-min intervals with Millonig's phosphate buffer, dehydration occurred at 25°C in a graded series of 50%, 70%, and 90% acetone, each step lasting at 10 min, followed by two washes with 100% acetone at 15-min intervals. The samples were then soaked and embedded in a 1:1 mix of acetone: resin for 12 h and 100% resin. Polymerization took place overnight at 37 °C, followed by another 12 h at 60 °C. Ultrathin sections of 100 nm were made from the specimens using an ultramicrotome and a diamond knife. After double staining with 3% uranyl acetate and lead nitrate, the specimens were examined and photographed using a Hitachi HT-7700 electron microscope.

Differentiation of iPSCs into NPCs

Generate NPCs by using the Monolayer Culture Protocol, according to the technical protocol of the STEMdiff™ SMADi Neural Induction Kit (STEMCELL Technologies Inc., Vancouver, Canada). On day 0, select dense clones and dissociate them into single cells using Gentle Cell Dissociation Agent (STEMCELL Technologies Inc., Vancouver, Canada). Seed the cells at a density of 2 x 10^5 cells/cm^2 onto Matrigel-coated 12-well or 24-well plates, and add sufficient volumes of STEMdiff™ Neural Induction Medium + SMADi + 10 μM Y-27632 (Selleck Chemicals, Houston, USA). From day 1 to day 7, perform a daily full-medium change with warm (37°C) STEMdiff™ Neural Induction Medium + SMADi until cultures are ready to be passaged (usually after approximately 7 days of culture).

RNA-Sequencing

Total RNA was extracted using Trizol reagent kit (Invitrogen, USA). RNA quality was assessed on an Agilent 2100 Bioanalyzer (Agilent Technologies, USA) and checked using RNase free agarose gel electrophoresis. Libraries were constructed by using NEBNext Ultra RNA Library Prep Kit for Illumina (NEB#7530, USA). The resulting cDNA library was sequenced using Illumina Novaseq6000. The reads were filtered by fastp (version 0.18.0) and mapped to the reference genome using HISAT2. The mapped reads of each sample were assembled by using StringTie v1.3.1. For each transcription region, a FPKM (fragment per kilobase of transcript per million mapped reads) value was calculated to quantify its expression abundance and variations, using RSEM software. Differentially expressed genes were identified by DESeq2 software with the parameter of false discovery rate (FDR) below 0.05 and absolute fold change≥2. GO or Pathway enrichment analysis provide all GO terms or pathways that significantly enriched in DEGs comparing to the genome background defined by hypergeometric test. The calculated p-value was gone through FDR Correction, taking FDR ≤ 0.05 as a threshold.

Supplementary Figures

Figure S1. **Generation of Pigk Purkinje cell-specific knockout mice.** (A) Image of Pigk-RNA *in situ* hybridization on the sagittal section of C57BL/6J mouse cerebellum from the website: https://mouse.brain-map.org/. (B) A schematic diagram for the *Pigk* knockout allele (cKO). The red arrowheads indicate loxP sites, and the thin black arrows indicate the primer binding site used for detecting the knockout band, with a size of 373 bp. (C) Gel electrophoresis image of PCR products from multiple tissues. The 567bp Cre band was detected in all tissues of cKO mice, while the knockout band was observed in the cerebellum of cKO mice. (D) Sanger sequencing indicated the knockout of exons 2 and 3 in the *Pigk* gene. The numbers indicate the relative positions of the bases in the *Pigk* genomic sequence, starting from the first base of exon 1.

Figure S2. **The female cKO mice exhibited progressive impaired motor coordination and ataxia, as shown in Fig. 1.** (A) Schematic diagram of the footprint experiment. Overlap is the distance between the front and hind paws; stride represents the distance between consecutive prints of the same paw, indicating the step length; sway is the vertical distance between a paw print and the contralateral paw print, representing the step width. Red pawprints represent the front paws, while blue represents the hind paws. (B–D) Overlap length (B), sway length (C), and stride length (D) between the forelimbs of Wt and cKO mice at 8 and 16 weeks. (E) Schematic diagram of the balance beam apparatus. Mice traverse from a 12-mm-wide wooden beam to a dark box located 100 cm away, positioned at a height of 50 cm above the ground. (F) The time spent crossing the balance beam increased in the cKO mice at 8 and 16 weeks. (G) Schematic diagram of the rotarod apparatus: The speed uniformly accelerates from 0 to 22 rpm within 1 min and then maintains a constant speed of 22 rpm for an additional 1 min. (H) The latency to fall and speed in the first fall of cKO mice decreased at 8 weeks. (B–H) N = 6 animals per genotype; Data are presented as

mean values + SEM; NS, no significance; *p < 0.05; **p < 0.01; ***p < 0.001; ****p < 0.0001 (Welch's *t*-test).

Figure S3. **Morphology of cerebellar and Purkinje cells in Wt and cKO mice at 4 weeks and 16 months.** (A-B) Gross view of brains from Wt and cKO mice at 4 weeks (A) and 16 months (B). (C-D) Immunostaining for calbindin on the sagittal sections of Wt and cKO mice at 4 weeks (C) and 16 months (D).

Figure S4. **KI iPSCs exhibit normal clone morphology.** (A) Sanger sequencing indicates that KI cells are homozygous for the *PIGK*: c.87dupT mutation. (B) The mRNA expression level of PIGK in KI iPSCs is comparable to that in WT iPSCs. N = 3 independent experiments. Data are presented as mean values + SEM; NS, no significance (Welch's *t*-test). (C) Western blot of PIGK indicates that PIGK protein is not detected in KI iPSCs. (D) Sanger sequencing for the CTF1, *MSMO1*, and *TLN2* genes indicates the absence of the predicted off-target effects in KI iPSCs. (E) KI iPSCs exhibit a normal karyotype. (F) Representative images of iPSC clones under bright-field microscopy show that the cloning morphology of KI iPSCs is indistinguishable from WT iPSCs. (G) Representative images of co-immunofluorescence staining for NANOG and SOX2. KI iPSCs showing no significant differences compared with WT iPSCs.

Figure S5. **Kira6 resulted in a reduction of apoptosis in KI iPSCs.** (A-B) Representative images of cleaved capapse3 staining (A) and quantification of the ratio of cleaved capapse3 ID to DAPI ID (B). N = 3 independent experiments; data are presented as mean values + SEM; NS indicates no significance; *p < 0.05; **p < 0.01; ***p < 0.01 (Welch's *t*-test).

Figure S6. **The KI iPSCs exhibited accelerated differentiation into NPCs** (A) Representative bright-field images of the differentiation process from iPSCs to NPCs on day1,3, 5, 7 and 8. Scale Bar=100μm. (B) Representative images of

immunofluorescence staining for SOX1 on day3, 5 and 7. (C) Quantification of the ratio of SOX1-positive cell numbers to total $DAPI^+$ cells in WT and KI cells on day3, 5 and 7. N = 3 independent experiments; data are presented as mean values + SEM; **p < 0.01; ***p < 0.001 (Welch's *t*-test).

Figure S1

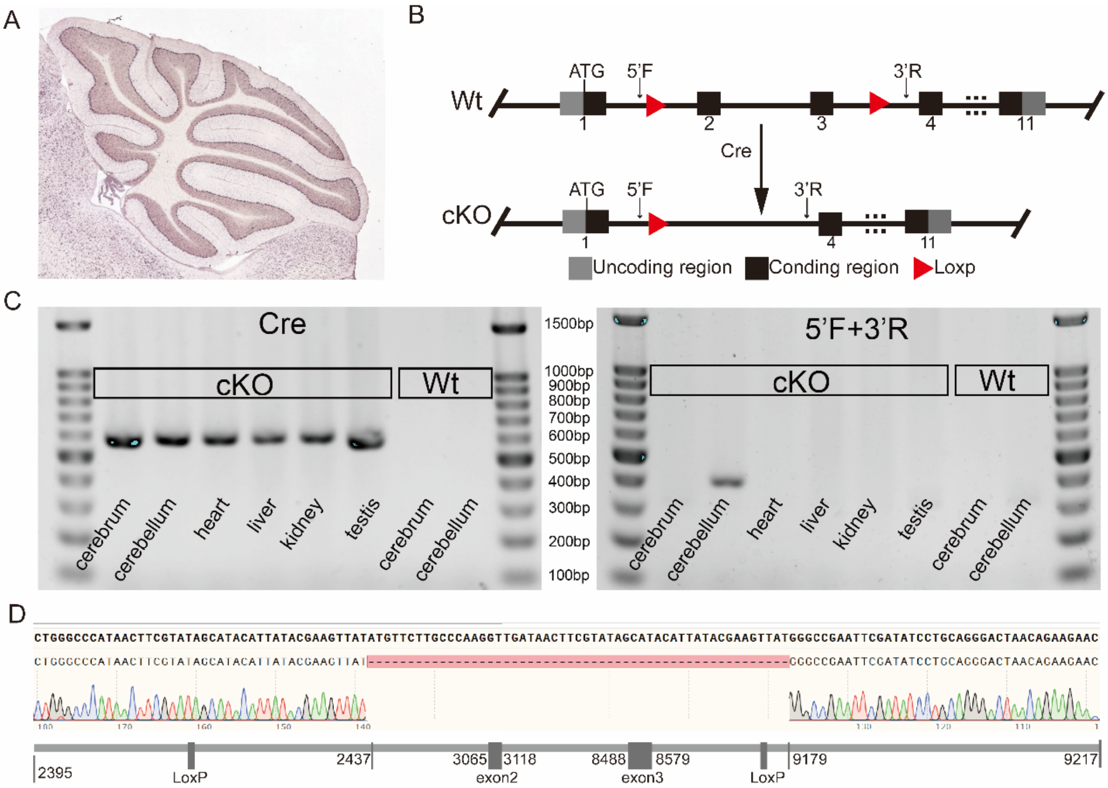

A
B
ATG
5'F
3'R
Wt
1
2
3
4
11
Cre
cKO
Uncoding region
Conding region
Loxp
C
Cre
cKO
Wt
cerebrum
cerebellum
heart
liver
kidney
testis
1500bp
1000bp
900bp
800bp
700bp
600bp
500bp
400bp
300bp
200bp
100bp
5'F+3'R
D
2395
LoxP
2437
3065
3118
exon2
8488
8579
exon3
9179
9217

Figure S2

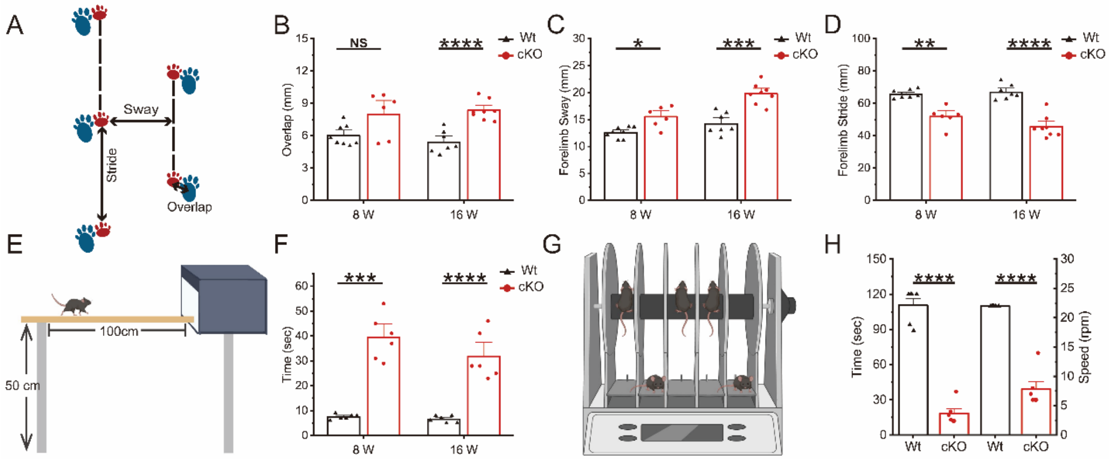

A
Sway
Stride
Overlap
B
NS
****
Wt
cKO
Overlap (mm)
8 W
16 W
C
*
***
Forelimb Sway (mm)
D
**
****
Forelimb Stride (mm)
E
100cm
50 cm
F
***
****
Time (sec)
G
H
Speed (rpm)
Wt
cKO

Figure S3

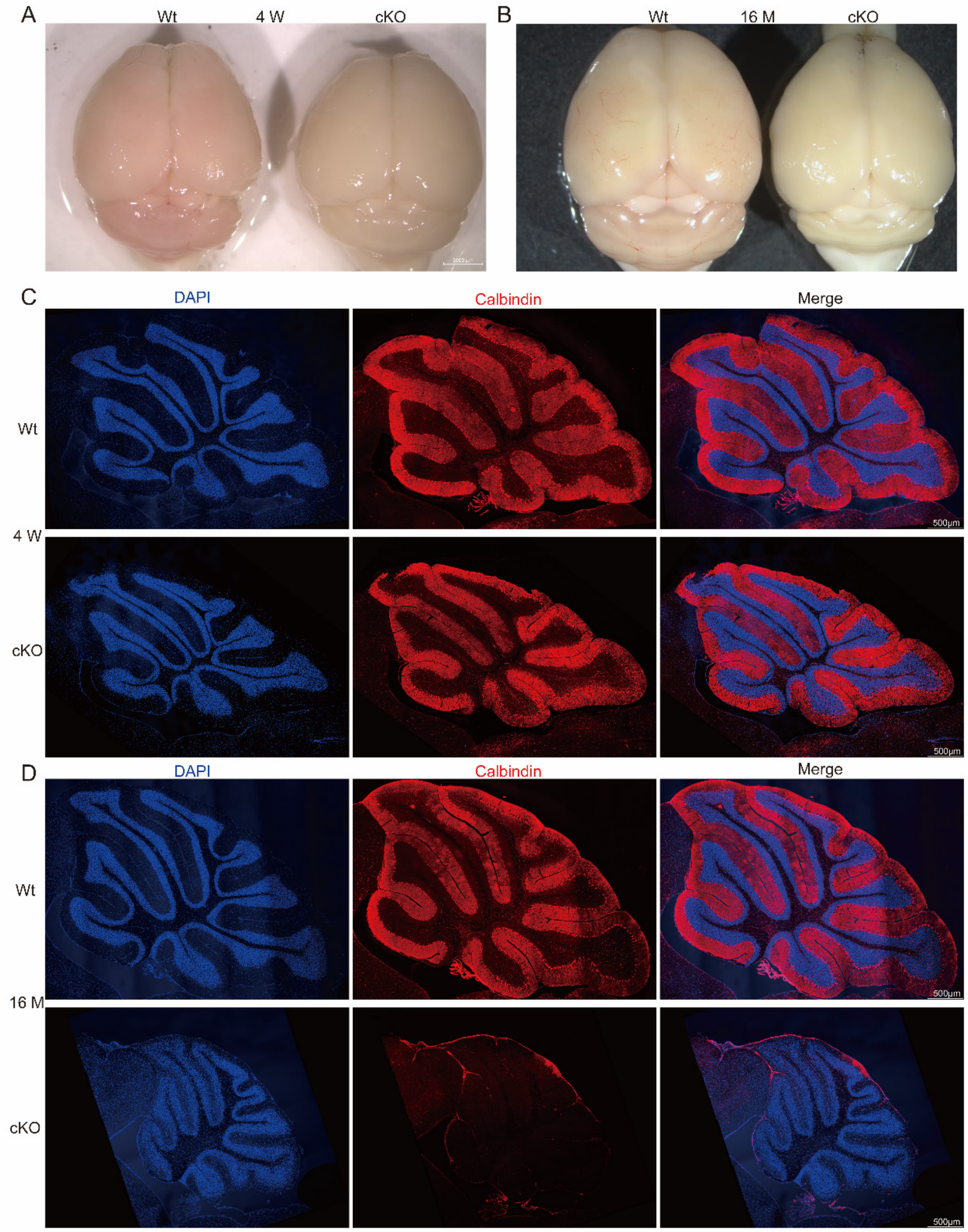
A
Wt
4 W
cKO
B
Wt
16 M
cKO
C
DAPI
Calbindin
Merge
Wt
4 W
cKO
500μm
500μm
D
DAPI
Calbindin
Merge
Wt
16 M
cKO
500μm
500μm

Figure S4

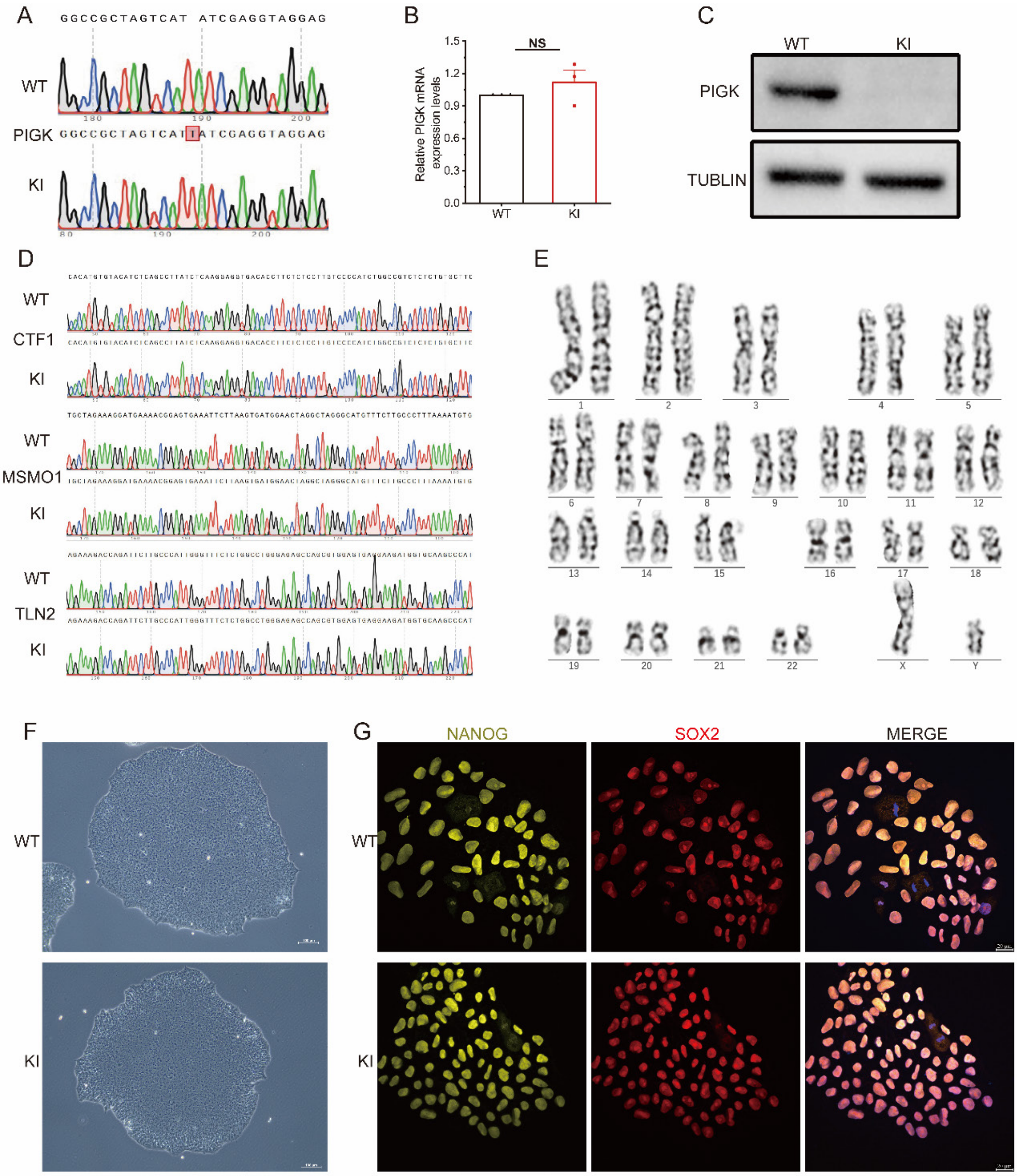

A
GGCCGCTAGTCAT ATCGAGGTAGGAG
WT
PIGK GGCCGCTAGTCATTATCGAGGTAGGAG
KI
B
NS
Relative PIGK mRNA expression levels
WT
KI
C
WT
KI
PIGK
TUBLIN
D
WT
CTF1
KI
WT
MSMO1
KI
WT
TLN2
KI
E
F
WT
KI
G
NANOG
SOX2
MERGE
WT
KI

Figure S5

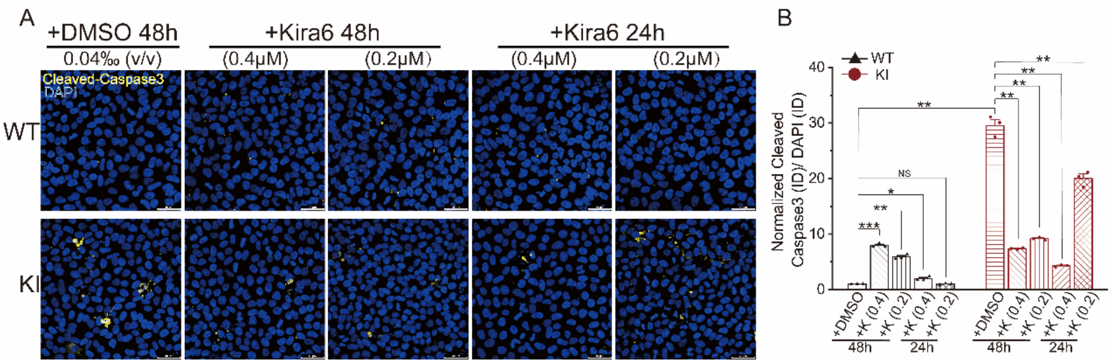
A
+DMSO 48h
0.04‰ (v/v)
+Kira6 48h
(0.4μM)
(0.2μM)
+Kira6 24h
(0.4μM)
(0.2μM)
Cleaved-Caspase3
DAPI
WT
KI
B
WT
KI
Normalized Cleaved Caspase3 (ID)/ DAPI (ID)
+DMSO
+K (0.4)
+K (0.2)
48h
24h
NS

Figure S6

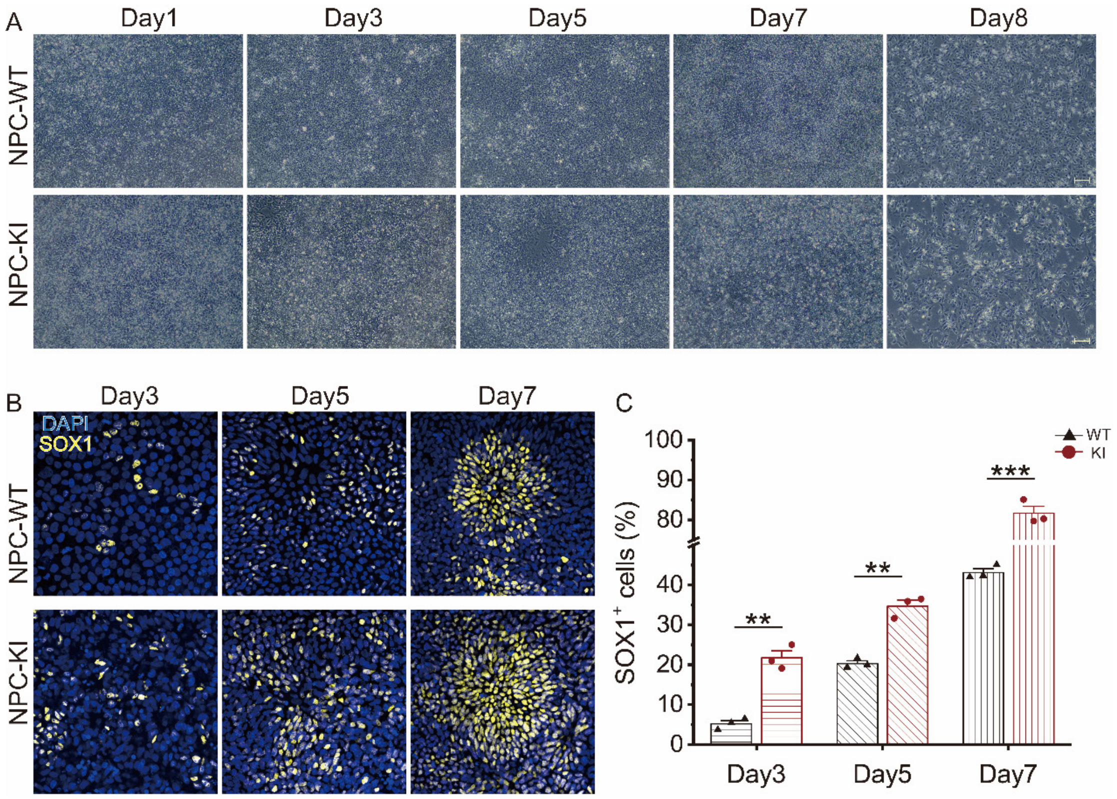
A
Day1
Day3
Day5
Day7
Day8
NPC-WT
NPC-KI
B
Day3
Day5
Day7
DAPI
SOX1
NPC-WT
NPC-KI
C
WT
KI
100
90
80
40
30
20
10
0
SOX1^{+} cells (%)
**
**
***
Day3
Day5
Day7

Movie S1 (separate file). Eight-week-old mouse's free activity.

Movie S2 (separate file). Sixteen-week-old mouse in the rotarod.

Supplementary Tables

Table S1. Primer name and sequence used in this study.

Primer name	5'–3'
Pigk-5'F	TCTGGGCTGATGGTTGGTAGG
Pigk-5'R	TCTGACACCCACAGATACATACAAGG
Pigk-3'R	GCAAACTCGCTGTAATAATCCCA
Pcp2-cre-F	ATTCTCGTGGAACTGGATGG
Pcp2-cre-R	GGACAGGTAATGGTTGTCTGG
PIGK-sgRNA-F	CACCGCGTGGCCGCTAGTCATATCG
PIGK-sgRNA-R	AAACCGATATGACTAGCGGCCACGC
ssODN	CTGTGTTGCTCTTGTCCTTCGGCAGCGTGGCCGCTAGTCATTATCGAGGTAGGAGTCAGGCTCATTTGACCACAGCCGGGA
PIGK-exon1-F	AAACCGCAGAGTTCTAAAGCC
PIGK-exon1-R	AACGCTGAAACAGACCAACA
PIGK-qPCR-F	GTGTCAAGAGGCTAGGTATTCC
PIGK-qPCR-R	AGCTGGTTTGGGATTTCTAGG
CTF1-3'UTR-F	CCCCCTTTACCCAGCTCTTG
CTF1-3'UTR-R	GATGCAGAGTAGCCTTCCCC
MSMO1-exon1-F	GAGGCAGGTTCCGAGGTT
MSMO1-exon1-R	CCCACTACTCCCAAAGCAC
TLN2-exon11-F	ACCAAAGACTCGGTGATG
TLN2-exon11-R	GAGAAGCAGTAGTGCCTGA

Table S2. Primary antibodies used in this study

Antibody name	Source	catalog	dilution
Mouse anti-Calbindin-D-28K	Sigma-Aldrich	cat#c9848	IF:1/200
Rabbit anti-Neun	GeneTex	cat#GTX132974	IF:1/200
Rabbit anti-GFAP	GeneTex	cat#GTX108711	IF:1/200
Rabbit anti-Iba1	abcam	cat#ab178846	IF:1/1000
Rabbit anti-NANOG	abcam	cat# ab109250	IF:1/200
Mouse anti-SOX2(L1D6A2)	Cell Signaling Technology	cat#4900	IF:1/200
Mouse anti-Ki-67(8D5)	Cell Signaling Technology	cat#9449	IF:1/200
Rabbit anti Cleaved Caspase-3(Asp175)	Cell Signaling Technology	cat#9661	IF:1/200
Rabbit anti-PIGK	abcam	cat#ab201693	WB:1/2,000
Rabbit anti Bip(C50B12)	Cell Signaling Technology	cat#3117	WB:1/1,000
Rabbit anti-Phospho-SAPK/JNK	Cell Signaling Technology	cat#4668	WB:1/1,000
Rabbit anti-SAPK/JNK	Cell Signaling Technology	cat#9252	WB:1/1,000
Rabbit anti-CHOP	proteintech	cat#15204-1-AP	WB:1/1,000
Rabbit anti-Phospho-eIF2α	Cell Signaling Technology	cat#3597	WB:1/1,000
Rabbit anti-eIF2α (D7D3)	Cell Signaling Technology	cat#5324	WB:1/1,000
Rabbit anti-SOX1	Cell Signaling Technology	cat#4194s	IF:1/200
Mouse anti-PAX6	Gene Tex	cat#GTX634863	IF:1/200

Original WB

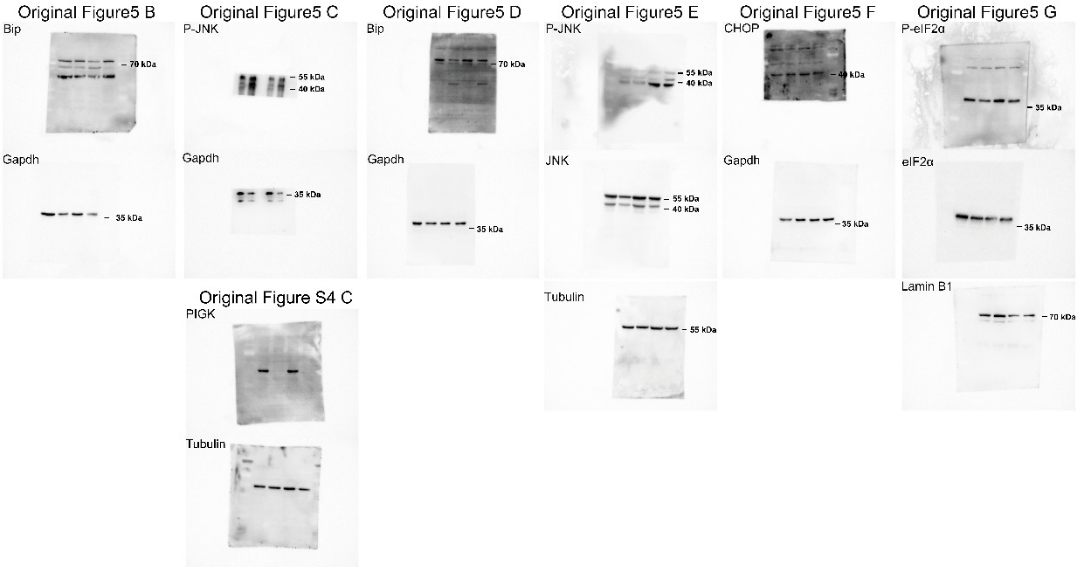
Original Figure5 B
Bip
70 kDa
Gapdh
35 kDa
Original Figure5 C
P-JNK
55 kDa
40 kDa
Gapdh
35 kDa
Original Figure5 D
Bip
70 kDa
Gapdh
35 kDa
Original Figure5 E
P-JNK
55 kDa
40 kDa
JNK
55 kDa
40 kDa
Tubulin
55 kDa
Original Figure5 F
CHOP
40 kDa
Gapdh
35 kDa
Original Figure5 G
P-eIF2α
35 kDa
eIF2α
35 kDa
Lamin B1
70 kDa
Original Figure S4 C
PIGK
Tubulin
